# Dr. Archambault on Paracentesis Thoracis of Children*Archiv Gen. de Med., Juillet, 1853.

**Published:** 1854-07

**Authors:** 


*Dr. Archambault on Paracentesis Thoracis in Children.**—In an inaugural thesis presented to the University of Paris during the past year, Dr. Archambault relates some instructive cases of this kind, which occurred in the practice of M. Trousseau, at the Hopital des Enfans Malades.

Case 1.—*Excessive pleuritic effusion, dating from eight days; operation; cure.*—The patient, a little boy, æt. 4 years, had been ill eight days. The illness began with a dry cough, oppressed breathing and slight fever, but without any shivering or pain in the side. When admitted (January 6th, 1852) the breathing was laborious, and 70 in the minute: pulse 120, and small, the left side of the chest prominent, not taking any part in the respiration, and everywhere dull on percussion, and without respiratory sounds,

*Archiv Gen. de Med., Juillet, 1853.

except immediately under the clavicle. The spleen was pushed considerable below the level of the false ribs, and the heart very much to the right of its proper position. The right side of the chest was healthy.

The day following, the respiration became more laborious, the surface dusky and cool, and asphyxia imminent, paracentesis thoracis was performed, under the sanction of MM. Trousseau, Blache, and Guersant, when a *litre* of yellowish transparent fluid was evacuated. Immediately afterward, the respiration became less frequent, the sound on percussion clear, the respiratory murmur audible, and the heart returned to its proper place.

On the 9th, the effusion returned to some extent, and a diuretic mixture with digitalis was ordered. On the day following there was vomiting and much depression, but the dullness diminished.

From the 12th to the 30th the patient progressed favorably, until the lung had recovered its healthy action, and all pleural rubbing had disappeared. Convalescence was retarded by a severe attack of measles, but in the end the child got perfectly well.

Case 2.—*Excessive sero-purulent effusion; operation; death.*—The patient was a little boy, aged two years, whose previous history was not ascertainable. When admitted into the hospital he was in a state of imimnent asphyxia; the face pale, the pulse small and excessively frequent, the respiration 68 in the minute. The left side of the chest was dilated, dull on percussion, and devoid of any kind of respiratory sound or vibration. The heart beat on the right side of the sternum. The left lung exhibited the signs of great congestion.

Paracentesis thoracis was performed on the 4th of June, 1852, at 11 A. M., and 420 grammes of sero-purulent liquid evacuated. Ten minutes afterward the symptoms were ameliorated in a marked manner, particularly the restlessness and anxiety. A loud *rale* became audible throughout the left side. Still the respiration continued at 50–54, and the pulsation at 132 in the minute.

Between the 5th and 8th, the improvement continued, the respiration falling to 44, and the pulsation to 120 in the minute.

On the 10th a relapse took place, the effusion became reproduced, and death took place on the 13th.

After death 300 grammes of sero-purulent flaky fluid were found in the left pleura, which pleura was everywhere covered with a thick, flocculent, false membrane. The two inferior thirds of the corresponding lung were carnified, and compressed against the vertebral column; the upper third and the other lung were congested and hepatized in several patches.

A third case is mentioned, of which no further particulars are given, than that the patient was a child, aged twelve years, who had labored under hydrothorax for fifteen days, and was cured by the operation.

